# Two Novel *Trichoderma* Species and their Antagonistic Activity against Sclerotia-producing Plant Pathogens

**DOI:** 10.1007/s00284-026-04769-6

**Published:** 2026-03-03

**Authors:** Jessica Rembinski, Lucas S. Sales, Phellippe A. S. Marbach, Jorge T. De Souza

**Affiliations:** 1 Department of Phytopathology, Federal University of Lavras (UFLA), Lavras, 37200-000 MG Brazil; 2 Evolutionary Biology laboratory, Federal University of Recôncavo da Bahia, 44380-000 Cruz das Almas-BA, Brazil

## Abstract

*Trichoderma* species are among the most employed fungal biocontrol agents in global agriculture. One mechanism of activity of these fungi is mycoparasitism, which is responsible for degrading the cell wall of other fungi through the production of enzymes such as chitinases and cellulases. This mechanism is capable of targeting resistant structures like sclerotia and chlamydospores produced by pathogens such as *Sclerotinia sclerotiorum* and *Stromatinia cepivora*. Several *Trichoderma* species have been reported as antagonists of these pathogens, but most described species were not explored for their antagonism against sclerotia-producing pathogens. The objective of this study was to characterize new species of the *Trichoderma* genus and evaluate their antagonistic potential against pathogens that produce sclerotia. The taxonomical novelties reported in this study are the description of two novel species, *T. variabile* and *T. littericola* from the Restinga ecosystem and *T. nordicum* is reported from Brazil, showing that this species has a broader distribution than previously thought. In addition to that, *T. nigricans* was shown to be an invalid species that should be considered a synonym of *T. nordicum* according to the rule of priority in taxonomical descriptions. These strains showed antagonistic potential against *S. sclerotiorum* and *S. cepivora in vitro*, especially *T. nordicum* and *T. littericola*, which were superior to *T. harzianum* from a commercial product. This study contributed to extend our knowledge on the diversity of *Trichoderma* species from the Restinga ecosystem and points out to their potential use in the biological control of plant pathogens that produce sclerotia.

**Supplementary Information:**

The online version contains supplementary material available at 10.1007/s00284-026-04769-6.

## Introduction

Sclerotia are melanized survival structures produced by microorganisms in the Kingdom *Fungi* and by *Myxomycetes* in the Kingdom *Amoebozoa* [1]. Within the Kingdom *Fungi* sclerotia appear to be produced only by representatives of the Phyla *Ascomycota* and *Basidiomycota* and were documented in at least 85 fungal genera in 20 orders [1]. These structures remain viable in soil for long periods of time and are resistant to chemicals, adverse conditions and biological degradation [2]. Sclerotia are very diverse in terms of morphology, composition and physiology. While some have a round shape, smooth surface, dark color and a differentiated ring encircling undifferentiated hyphae, such as the ones produced by *Agroathelia rolfsii*, others have an irregular shape, undifferentiated ring and lighter color, such as the ones produced by *Rhizoctonia solani*. Sclerotia produced by *Macrophomina phaseolina*, *Stromatinia cepivora* and *Verticillium dahliae* are smaller than 1 mm, but the ones produced by *Polyporus mylittae* may reach up to 40 cm in diameter [3].

The species *Sclerotinia sclerotiorum*, *S. cepivora*, *R. solani*, *A. rolfsii* and *M. phaseolina* are among the most harmful sclerotia-producing pathogenic fungi in agriculture worldwide [4–7]. These pathogens are hard to manage due to their survival capabilities through sclerotia associated with successive monocultures in the same area and the lack of efficient soil fumigation alternatives.

Biological control with mycoparasites such as *Trichoderma* is an interesting strategy to decrease the number of sclerotia in soil and thereby control these pathogenic fungi. All *Trichoderma* species are known for having the ability to parasitize other fungi, a characteristic that was inherited from the ancestor of this genus [8]. The species *T. hamatum*, *T. viride*, *T. aureoviride*, *T. harzianum*, *T. koningii*, *T. pseudokoningii*, *T. longibrachiatum* and *T. asperellum* were reported as parasites of sclerotia [9–12]. This mycoparasitic capacity varies among species and strains of the same species. *Trichoderma* is highly speciose, currently containing approximately 400 described species [13] and most of them are still unexplored for potential biotechnological applications, such as sclerotia mycoparasitism and control of pathogenic fungi.

During our studies on the fungal diversity of the Brazilian Restinga ecosystem we isolated 26 *Trichoderma* strains, 22 were found to be related to *T. spirale* and were reported in another study done by our research group [14]. The other four strains represent two novel species of *Trichoderma* and will be illustrated and described in this study. Furthermore, the novel species were tested for their potential to parasitize sclerotia of fungal pathogens. We studied a Brazilian strain of a recently described species, *T. nordicum* [15] and made observations on its taxonomy, mycoparasitic activity and distribution.

## Materials and Methods

### Fungal Strains and Growth Conditions

The fungi used in this study (Table 1) included four strains of the novel *Trichoderma* species isolated from the Restinga ecosystem: URMicro 11718, URMicro 11720, URMicro 11721 and URMicro 11723, and one strain of *T. nordicum*, URMicro 11722, isolated from the Amazonian region of Brazil. *Trichoderma harzianum* H1 was isolated from a commercial product recommended to control *S. sclerotiorum* in Brazil and was used for comparison purposes in the experiments to study sclerotia parasitism, but was not included in our phylogenetic analyses. The sclerotia-producing pathogen *S. cepivora* strain JT101 was obtained from an infected garlic plant in the municipality of São Gotardo, MG, Brazil and *S. sclerotiorum* strain JT205 was isolated from an infected soybean plant at the municipality of Lavras, MG, Brazil. All fungal strains were routinely grown on PDA (Potato Dextrose Agar). *Trichoderma* and *S. sclerotiorum* were grown at 25 °C and *S. cepivora* at 17 °C. Sclerotia were produced on plates incubated at the above temperatures after 14 days. All strains were preserved at −20 °C in the working collection of the Molecular Phytopathology Laboratory and the *Trichoderma* strains were also deposited in the URMicro culture collection at −80 °C, Federal University of Lavras, Brazil, which is registered with the World Federation for Culture Collections and can make the strains available for research purposes upon request (Table 1).

**Table 1:** Accession numbers of sequences used in this study to infer phylogenies. The newly described species are shown in bold and the type strains are indicated with a superscript T

Species	Clade	Strain	Accession numbers		
			tef1	rpb2	cal1
*T. applanatum*	Hypocreanum	7792 ^T^	KJ634759	KJ634726	-
*T. austriacum*	Hypocreanum	CBS 122494 ^T^	FJ860619	FJ860525	-
*T. confluens*	Hypocreanum	9649 ^T^	KT001959	KT001964	-
*T. decipiens*	Hypocreanum	CBS 121307 ^T^; GJS 91–101	FJ860635	DQ835520	-
*T. phellinicola*	Hypocreanum	CBS 119283 ^T^	FJ860672	FJ860569	-
*T. pulvinatum*	Hypocreanum	CBS 121279 ^T^	FJ860683	FJ860577	-
*T. protopulvinatum*	Hypocreanum	CBS 739.83 ^T^	FJ860679	DQ835463	-
*T. sulphureum*	Hypocreanum	CBS 119929 ^T^	FJ860710	FJ179620	-
***T. variabile***	Hypocreanum	URMicro 11718	OR762225	OR762229	OR762234
***T. variabile***	Hypocreanum	URMicro 11720	OR762224	OR762228	OR762233
***T. variabile***	Hypocreanum	**URMicro 11721** ^**T**^	OR762226	OR762230	OR762235
*T. victoriense*	Hypocreanum	GJS 99–200 ^T^	DQ835500	DQ835517	-
*T. alboviride*	Longibrachiatum	TC916; HMAS 247224 ^T^	MF371230	MF371215	-
*T. andinense*	Longibrachiatum	GJS 90–140 ^T^	AY956321	AY956321	JN175412
*T. aquatica*	Longibrachiatum	YMF 1.04625 ^T^	MK775507	MK775512	-
*T. awajun*	Longibrachiatum	CP24_7 ^T^	MW480138	MW480147	-
*T. awajun*	Longibrachiatum	CP24_8	MW480139	MW480148	-
*T. bissettii*	Longibrachiatum	P3572	PV081282	PV081389	-
*T. bissettii*	Longibrachiatum	P3606	PV081290	PV081397	-
*T. bissettii*	Longibrachiatum	SZMC 25718	-	-	MN650686
*T. bissettii*	Longibrachiatum	SZMC 1012	-	-	MN641032
*T. bissettii*	Longibrachiatum	SZMC 1158	-	-	MN641033
*T. bissettii*	Longibrachiatum	SZMC 1767	-	-	MN650683
*T. britdaniae*	Longibrachiatum	K 89878 ^T^	JQ685865	JQ685881	-
*T. caeruleimontis*	Longibrachiatum	PPRI 23903 ^T^	-	-	MF355410
*T. caeruleimontis*	Longibrachiatum	Tri 43	-	-	MF355412
*T. caeruleimontis*	Longibrachiatum	Tri 62	-	-	MF355411
*T. euskadiense*	Longibrachiatum	CBS 130013 ^T^	KJ665492	KJ665269	-
*T. flagellatum*	Longibrachiatum	PPRC-ET58; GJS 10–164 ^T^	FJ763184	KR297247	-
*T. gillesii*	Longibrachiatum	GJS 00–72 ^T^	JN175583	JN175527	JN175409
*T. gracile*	Longibrachiatum	GJS 10–263 ^T^	JN175598	JN175547	JN175427
*T. konilangbra*	Longibrachiatum	GJS 96–147	AY937425	KJ665284	-
*T. longibrachiatum*	Longibrachiatum	CBS 816.68 ^T^	AY865640	DQ087242	EU401459
*T. longibrachiatum*	Longibrachiatum	DAOM 166989	EU338335	EU338339	-
*T. longibrachiatum*	Longibrachiatum	ATCC 18648 ^T^	AY937412	HQ260615	MBDJ01001225
*T. longibrachiatum*	Longibrachiatum	GJS 01–121	JN175564	JN175507	JN175387
*T. longibrachiatum*	Longibrachiatum	GJS 08–198	JN175565	JN175508	JN175388
*T. longibrachiatum*	Longibrachiatum	GJS 04–53	JN175568	JN175512	-
*T. longibrachiatum*	Longibrachiatum	GJS 07–21	JN175569	JN175513	-
*T. longibrachiatum*	Longibrachiatum	S328	JQ685867	KJ665291	-
*T. longibrachiatum*	Longibrachiatum	DAOM 167674	EU280046	KJ842212	-
*T. longibrachiatum*	Longibrachiatum	LESF009	KT279040	KT278975	-
*T. longibrachiatum*	Longibrachiatum	TLC4	MT069949	MT069960	-
*T. longibrachiatum*	Longibrachiatum	TLS6	MT069951	MT069959	-
*T. longibrachiatum*	Longibrachiatum	Tloum3	MT081437	MT118251	-
*T. longibrachiatum*	Longibrachiatum	HL167	MZ241241	MZ241240	-
*T. longibrachiatum*	Longibrachiatum	HNDF-G-2	MW504035	MZ357387	-
*T. longibrachiatum*	Longibrachiatum	HNDF-G-4	MW504037	MZ357389	-
*T. longibrachiatum*	Longibrachiatum	HNDF-G-5	MW504038	MZ357390	-
*T. longibrachiatum*	Longibrachiatum	JZBQT8Z7	ON649943	ON649996	-
*T. longibrachiatum*	Longibrachiatum	SC5	ON808971	ON815270	-
*T. longibrachiatum*	Longibrachiatum	D612	ON934353	ON934388	-
*T. longibrachiatum*	Longibrachiatum	E105	ON934356	ON934391	-
*T. longibrachiatum*	Longibrachiatum	E106	ON934357	ON934392	-
*T. longibrachiatum*	Longibrachiatum	E356	ON934360	ON934395	-
*T. longibrachiatum*	Longibrachiatum	E357	ON934361	ON934396	-
*T. longibrachiatum*	Longibrachiatum	E473	ON934362	ON934397	-
*T. longibrachiatum*	Longibrachiatum	E475	ON934363	ON934398	-
*T. longibrachiatum*	Longibrachiatum	KUFA0409	OP132641	OP132653	-
*T. longibrachiatum*	Longibrachiatum	KUFA0410	OP132642	OP132654	-
*T. longibrachiatum*	Longibrachiatum	CHE-CNRCB-334	OP410938	OP410935	-
*T. longibrachiatum*	Longibrachiatum	593	OR548051	OR548102	-
*T. longibrachiatum*	Longibrachiatum	539	OR548074	OR548106	-
*T. longibrachiatum*	Longibrachiatum	FAVF335	PP195794	PP195792	-
*T. longibrachiatum*	Longibrachiatum	FAVF340	PP195795	PP195793	-
*T. longibrachiatum*	Longibrachiatum	CEN1281	ON101480	PP854164	ON241222
*T. longibrachiatum*	Longibrachiatum	CEN1562	ON101481	PP854165	-
*T. longibrachiatum*	Longibrachiatum	CHE-CNRCB 1292	PV555431	PV555433	-
*T. longibrachiatum*	Longibrachiatum	CHE-CNRCB 1293	PV555432	PV579007	-
*T. longibrachiatum*	Longibrachiatum	CHE-CNRCB 1294	PV579006	PV579008	-
*T. longibrachiatum*	Longibrachiatum	IAA 1	-	-	EU401444
*T. longibrachiatum*	Longibrachiatum	DAOM 231258	-	-	EU401480
*T. longibrachiatum*	Longibrachiatum	DAOM 231259	-	-	EU401479
*T. longibrachiatum*	Longibrachiatum	PPRC SS8	-	-	EU401486
*T. longibrachiatum*	Longibrachiatum	CPK 744	-	-	JN182288
*T. longibrachiatum*	Longibrachiatum	Tp-05	-	-	KC967327
*T. longibrachiatum*	Longibrachiatum	Tp-09	-	-	KC967334
*T. longibrachiatum*	Longibrachiatum	IMI 287096	-	-	EU401462
*T. longibrachiatum*	Longibrachiatum	IMI 291014	-	-	EU401463
*T. longibrachiatum*	Longibrachiatum	ATCC 201044	-	-	EU401468
*T. longibrachiatum*	Longibrachiatum	ATCC 208859	-	-	EU401469
*T. longibrachiatum*	Longibrachiatum	CBS 446.95	-	-	EU401470
*T. longibrachiatum*	Longibrachiatum	CNM-CM 382	-	-	EU401472
*T. longibrachiatum*	Longibrachiatum	CNM-CM 2277	-	-	EU401475
*T. longibrachiatum*	Longibrachiatum	CNM-CM 1798	-	-	EU401473
*T. longibrachiatum*	Longibrachiatum	Tl-03	-	-	KC967339
*T. longibrachiatum*	Longibrachiatum	Tl-04	-	-	KC967340
*T. longibrachiatum*	Longibrachiatum	Tl-08	-	-	KC967344
*T. longibrachiatum*	Longibrachiatum	Tl-10	-	-	KC967346
*T. longibrachiatum*	Longibrachiatum	Tl-12	-	-	KC967348
*T. longibrachiatum*	Longibrachiatum	Tl-14	-	-	KC967350
*T. longibrachiatum*	Longibrachiatum	SZMC 23386	-	-	MN650682
*T. longibrachiatum*	Longibrachiatum	UAMH 7955	-	-	EU401464
*T. longibrachiatum*	Longibrachiatum	UAMH 9515	-	-	EU401466
*T. longibrachiatum*	Longibrachiatum	CECT 20105	-	-	EU401476
*T. longibrachiatum*	Longibrachiatum	CECT 2412	-	-	EU401491
*T. longibrachiatum*	Longibrachiatum	TUB F-363	-	-	EU401449
*T. longibrachiatum*	Longibrachiatum	TUB F-1036	-	-	EU401454
*T. longibrachiatum*	Longibrachiatum	TUB F-828	-	-	EU401450
*T. longibrachiatum*	Longibrachiatum	Kazan 8	-	-	EU401478
*T. longibrachiatum*	Longibrachiatum	TH2	-	-	KU554610
*T. longibrachiatum*	Longibrachiatum	Y 19	-	-	EU401457
*T. longibrachiatum*	Longibrachiatum	GEV 3550	-	-	JASTZJ010000008
*T. longibrachiatum*	Longibrachiatum	R6902	-	-	JAXQJK010000013
*T. longibrachiatum*	Longibrachiatum	M236	-	-	ON157427
*T. novae-zelandiae*	Longibrachiatum	GJS 81–265 ^T^	AY937448.2	JN133563	JN175406
*T. odoratum*	Longibrachiatum	HMAS 271354 ^T^	KT224463	KT224468	-
*T. orientale*	Longibrachiatum	GJS 88 − 81 ^T^	EU401581	JN175522	EU401448
*T. orientale*	Longibrachiatum	TUB F-831	-	-	EU401451
*T. orientale*	Longibrachiatum	TUB F-837	-	-	EU401452
*T. orientale*	Longibrachiatum	CPK 3598	-	-	JQ425719
*T. orientale*	Longibrachiatum	GJS 04–333	-	-	JN175399
*T. orientale*	Longibrachiatum	GJS 04–316	-	-	JN175400
*T. orientale*	Longibrachiatum	GJS 10–230	-	-	JN175403
*T. orientale*	Longibrachiatum	GJS 04–321	-	-	N175397
*T. orientale*	Longibrachiatum	GJS 10–253	-	-	JN388899
*T. orientale*	Longibrachiatum	GJS 91–157	-	-	EU401461
*T. orientale*	Longibrachiatum	BMU09741	-	-	MN447669
*T. orientale*	Longibrachiatum	DIS 270 F	-	-	JN175401
*T. orientale*	Longibrachiatum	SZMC IM1	-	-	EU401493
*T. orientale*	Longibrachiatum	SZMC IM2	-	-	EU401494
*T. orientale*	Longibrachiatum	UAMH 9573	-	-	EU401467
*T. orientale*	Longibrachiatum	CECT 2606	-	-	EU401477
*T. parareesei*	Longibrachiatum	TUB F-1066 ^T^	GQ354353	HM182963	GQ354288
*T. patella*	Longibrachiatum	GJS 91–141^T^	KJ665630	KJ665323	-
*T. pinnatum*	Longibrachiatum	GJS 04–100 ^T^	JN175571	JN175515	JN175395
*T. pinnatum*	Longibrachiatum	GJS 02–120	JN175516	JN175572	-
*T. pinnatum*	Longibrachiatum	LS029-3	OK652543	OK652544	ON890478
*T. pluripenicillatum*	Longibrachiatum	YMF 1.06198 ^T^	MT070159	MT070160	-
*T. pseudobritdaniae*	Longibrachiatum	HMAS 271355 ^T^	KT224462	KT224466	-
*T. pseudokoningii*	Longibrachiatum	DAOM 167678 ^T^	KJ713204	KJ842214	JN175416
*T. reesei*	Longibrachiatum	DAOM 167654 ^T^; CBS 383.78	KJ713193	HM182969	JN180917
*T. saturnisporiopsis*	Longibrachiatum	S19 ^T^	JQ685869	JQ685885	JQ349444
***T. littericola***	Longibrachiatum	**URMicro 11723** ^**T**^	OR762227	OR762231	OR762236
*T. solani*	Longibrachiatum	GJS 08–81 ^T^	JN175597	JN175546	JN175426
*T. tremelloides*	Longibrachiatum	CBS 121140 ^T^	FJ860714	FJ860603	-
*Trichoderma* sp	Longibrachiatum	CFCC 80875 A	PV755843	PV617129	-
*Trichoderma* sp	Longibrachiatum	CFCC 82906 A	PV755851	PV617137	-
*Trichoderma* sp	Longibrachiatum	CFCC 88967 A	PV755878	PV617166	-
*Trichoderma* sp	Longibrachiatum	CFCC 89492 A	PV755881	PV617169	-
*Trichoderma* sp.	Longibrachiatum	PF4103 ctg 22	-	-	JAMAKC010000022
*Trichoderma* sp.	Longibrachiatum	CBS 243.63	-	-	EU401460
*T. nordicum*	Viride	URMicro 11722	HG325833	OR762232	-
*T. nordicum*	Viride	WT 13001 ^T^	MH287501	MH287502	-
*T. strigosum*	Viride	DAOM 166121 ^T^	EU280019	AF545556	JN133544

### Morphological Characterization

Morphological studies were done on corn meal agar dextrose (CMD), synthetic nutrient agar (SNA) and potato dextrose agar (PDA) according to [15–18]. Growth rates were determined at 15, 20, 25, 30 and 35 °C with a 12/12 h light/dark photoperiod using near UV halogen lamps (300–400 nm) [18]. All experiments were installed with four replicates and were done two times.

The micromorphological characteristics were analyzed on structures produced on SNA after three days of incubation at 25 °C. Images were captured in a microscope with fluorescence using the Apotome system and confocal laser (Carl Zeiss) and a scanning electron microscope STEM-FEG model Clara Tescan. Measurements were performed in at least 50 structures of each type, including conidia, phialides, conidiophores and chlamydospores.

### Molecular Characterization

Extraction of DNA was done according to the CTAB protocol [19] with mycelium produced with cultures grown for 3–5 days in potato dextrose broth (PDB) (HiMedia) at 25 °C. The primers and amplification conditions for translation elongation factor 1-alpha (*tef1*), RNA polymerase subunit II (*rpb2*) and calmodulin (*cal1*) are described in [20–22]. PCR products were sequenced by the Sanger method in an ABI 3100 according to the manufacturer’s instructions (Applied Biosystems). DNA sequences were assembled with Sequencher v. 5.1 (Gene Codes).

The sequences were compared with the ones deposited in public databases with the program BlastN in the NCBI platform. Multiple alignments were done with the program MAFFT [23] and edited in the program Mega 7.0 [24]. The phylogenetic analyses were done in the CIPRES portal [25] with the Maximum Likelihood (ML) method. Evolutionary models were selected with the program Jmodeltest 2.1.10 [26] using the corrected Akaike information criterion (AICc). The ML analyses were done with RAxML v.1.3 [27] with rapid bootstrapping parameters and 1,000 resamplings. Bayesian inferences were done using the program MrBayes version 3.2 [28] with four Monte Carlo Markov Chains (MCMC), one hot, three cold in one million generations with samplings at every one thousand generations. At the burning phase, 25% of the trees were excluded and the posterior probabilities (PP) were estimated by the remaining 75% of the trees. Trees were visualized with the program FigTree v1.4.4 [29].

### Antagonistic Activity of *Trichoderma*

The antagonism of *Trichoderma* was evaluated in bioassays to detect the secretion of diffusible and volatile organic compounds and also their capacity to parasitize sclerotia of *S. sclerotiorum* and *S. cepivora*. To test diffusible and volatile compounds, mycelial plugs of 5 mm diameter of each pathogen and of the different *Trichoderma* strains were placed in opposite positions at 0.5 cm from the edge of 9-cm diameter Petri plates containing PDA medium. Volatile compounds were evaluated in plastic plates split into two compartments (Axygen). Controls were plates containing each of the pathogens alone. Plates were incubated at 17 °C for approximately 7 days, when the controls reached the edge of the plate. This temperature was used to favor sclerotial germination and growth of the pathogens. The diameter of the pathogen colonies was determined with a caliper and percentage of inhibition in relation to the control was calculated for each of the *Trichoderma* strains [14]. The treatments were strains of the newly described species, *T. variabile* sp. nov. and *T. littericola* sp. nov., *T. harzianum* and *T. nordicum*. The experiments were installed with five replicates and were done twice.

Evaluation of sclerotia parasitism was performed by depositing a drop (~ 8 uL) of a suspension containing 2 × 10^7^ conidia/mL on the surface of each sclerotia of *S. sclerotiorum* and *S. cepivora*, which were produced on petri dishes with PDA medium. The sclerotia of *S. cepivora* were placed on the surface of sterile filter paper (Whatman no. 2) moistened with sterile water and incubated at 17 °C for 14 days. Sclerotia of *S. sclerotiorum* were placed on the surface of gerbox boxes containing 150 g of sterilized soil maintained approximately at the field capacity and incubated at 17 °C for 15 days. Each treatment, which were the *Trichoderma* strains mentioned above, was installed in five replicates of 10 sclerotia of each pathogen and the whole experiment was done twice. After the incubation period, sclerotia were surface sterilized with 70% ethanol for 1 min and 1% sodium hypochlorite for 2 min and three rinses with sterile water and plating on PDA medium and incubation at 17 °C. Sclerotia that showed mycelial growth were considered viable and the percentage of dead sclerotia was determined by observing the colonies of the pathogens and/or *Trichoderma* emerging from the sclerotia.

The data on antagonism of the different *Trichoderma* species against the sclerotia-producing fungi were submitted to statistical analysis, including the Shapiro-Wilk normality test, analysis of variance and multiple mean comparison with the Scott-Knott test at 5% probability in the program R using the package Scott-Knott version 1.3–2.3 [30].

## Results

### Phylogeny and Delimitation of the New Species

**Fig. 1:**
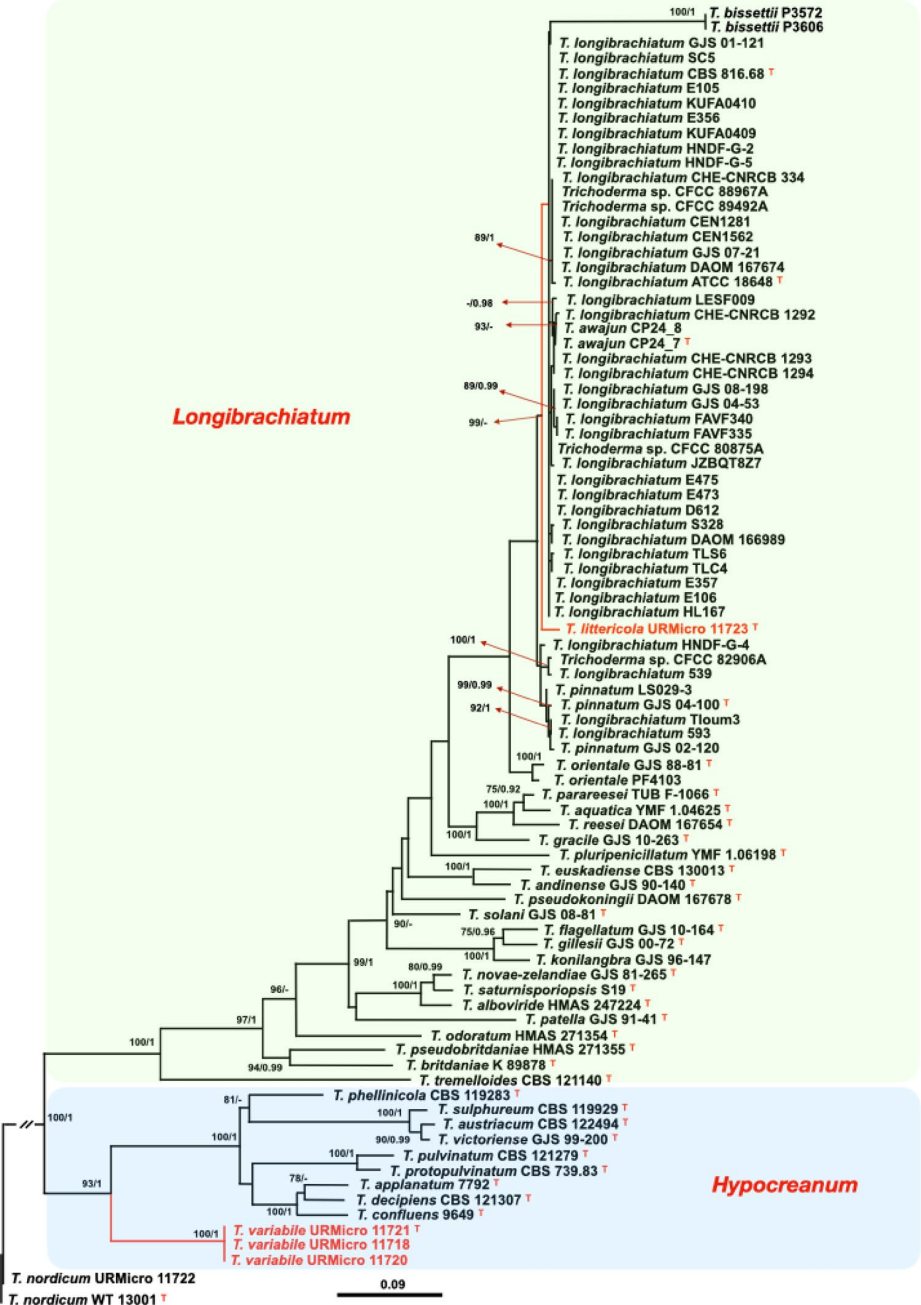
Phylogenetic tree inferred with the maximum likelihood (ML) and Bayesian inference (BI) methods with combined sequences of *tef1* and *rpb2* genes. The ML tree was inferred with two partitions, 617 bp of *tef1* and 544 bp of *rpb2* and the evolutionary model TPM3uf + I+G. The species *Trichoderma nordicum* strains URMicro 11722 and WT 13001 were used as outgroups. The numbers on the nodes represent the bootstraps (1000 resamplings) in ML analysis followed by the posterior probabilities (PP) in the Bayesian analysis. A dash means that the bootstrap or PP values are not significant according to the following reference values for significance: >70% for bootstrap and > 0.95 for PP. The species described as novel in this study are shown in red. The clades of *Trichoderma* are shown in different background colors. The type strains of the described species are indicated with a superscript T. The scale indicates the number of substitutions per site

Sequences of the genes *tef1*, *rpb2* and *cal1* (Table 1) were used to reconstruct phylogenetic trees with the methods maximum likelihood (ML) and Bayesian inference (BI). The individual and concatenated sequences of *tef1* and *rpb2*, and sequences of *cal1* supported *Trichoderma variabile* as a novel species with the maximum level of support in all cases (Fig. 1, Figs. S1, S2 and S3). On the other hand, *T. littericola* was well supported by ML in all the datasets, but not by the BI analysis in the combined *tef1* and *rpb2* and in *tef1* trees (Fig. 1, Fig. S1). Nevertheless, it appeared as a well-defined branch in all datasets.

BlastN analyses of *tef1* sequences of the type strain of *Trichoderma variabile* sp. nov. URMicro 11721 revealed that it was closest to the type strain of *T. confluens*, with a 93.9% identity (accession number KT001959) and 76% coverage; its *rpb2* sequence was 89.1% identical to the *rpb2* of *T. taxi* (DQ859032) with 99% coverage and *cal1* was 80.3% identical to *T. protrudens* (JN133540) with 80.3% coverage.

The type strain of *T. littericola* sp. nov. URMicro 11723 had a *tef1* sequence 91.9% identical to the *tef1* of *T. reesei* (CP040224) with 98% coverage; its *rpb2* was 98.2% identical to the *rpb2* of *T. longibrachiatum* (HQ260615) with 94% coverage; and its *cal1* was 96% identical to *T. pinnatum* (JN175395) with 98% coverage.

Strain URMicro 11722 of *T. nordicum*, which is not a new species, had its *tef1* 99.8% (93% coverage) identical to the type strain WT 13001 of *T. nordicum* (accession number MH287501) and 99.4% identical (98% coverage) to the type strain (T32781) of *T. nigricans* (accession OP357974). Similarly, its *rpb2* sequence was 100% (91% coverage) identical to the type strain of *T. nordicum* (MH287502) and 100% (75% coverage) identical to the type strain of *T. nigricans* (accession OP357959).

### Antagonism of *Trichoderma* against Sclerotia-producing Pathogens

**Fig. 2:**
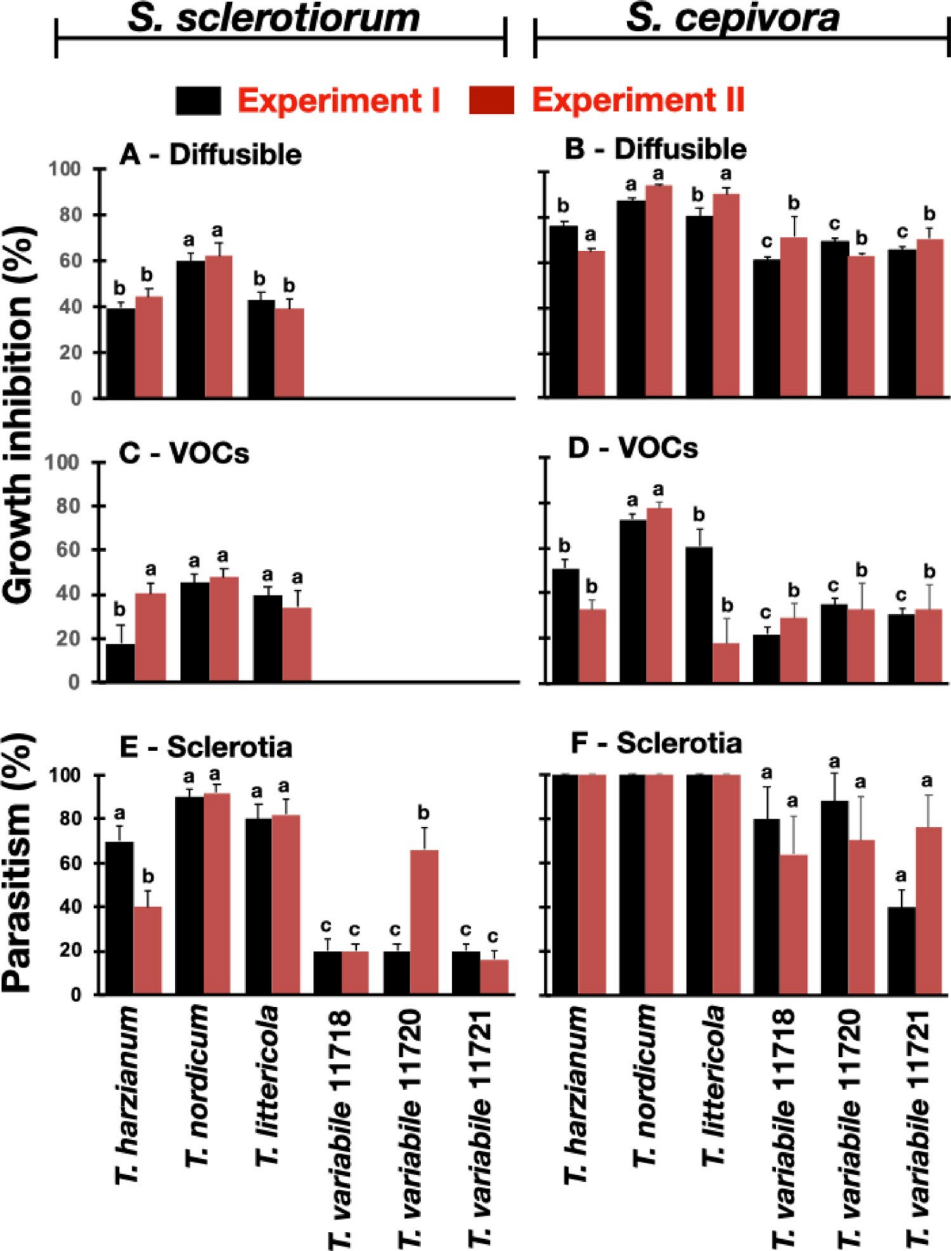
Antagonism of different *Trichoderma* strains to *Sclerotinia sclerotiorum* and *Stromatinia cepivora* in in vitro experiments done twice. **A-D** Mycelial growth of *S. sclerotiorum* **(A**,** C)** and *S. cepivora* **(B**,** D)** influenced by diffusible compounds produced by strains of *Trichoderma* in the culture medium **(A**,** B)** or by organic volatile compounds (VOCs) released in the headspace of sealed plates split into two compartments **(C**,** D)**. **E**,** F** Parasitism of sclerotia of *S. sclerotiorum* **(E)** and *S. cepivora* **(F)**. Data are means of five replicates per treatment per experiment and are shown in percentage of mycelial inhibition or in percentage of sclerotia parasitism. The experiments were done in plates and mycelial growth was measured when the control (pathogens alone) reached the edge of the plates. Parasitism was determined by inoculating sclerotia with spore suspensions of *Trichoderma* and placing them on the surface of sterilized soil or filter paper, respectively for sclerotia of *S. sclerotiorum* and *S. cepivora*. The number of parasitized sclerotia was determined after incubation for two weeks at 17 °C. Means followed by the same letter are not significantly different according to the Scott-Knott test at 5% probability. Comparisons should be done among means of treatments in the same experiment, not between experiments. Treatments without variance (0 or 100%) were removed from the statistical analysis

The antagonistic potential of six strains of *Trichoderma* was tested, including strains of the species being described in this study, *T. variabile* sp. nov. and *T. littericola* sp. nov., one strain of *T. harzianum* isolated from a commercial product and one of *T. nordicum* (Fig. 2). The antagonism of these strains was studied against *S. sclerotiorum* and *S. cepivora* considering three mechanisms of activity, production of diffusible compounds, release of volatile organic compounds and sclerotia parasitism. Mycelia and sclerotia of *S. cepivora* were more sensitive to *Trichoderma* than the sclerotia and mycelia of *S. sclerotiorum* (Fig. 2). The mechanism that showed the strongest activity against the pathogens was sclerotia parasitism. The new species *T. variabile* sp. nov. showed the poorest antagonistic activity in all the three mechanisms against both pathogens, whereas, in general, *T. nordicum* showed the strongest activity, surpassing *T. harzianum* in most bioassays. For example, *T. nordicum* parasitized over 90% of the sclerotia of *S. sclerotiorum*, while parasitism by *T. harzianum* was approximately 55% (Fig. 2E). The newly described species *T. littericola* sp. nov. showed a spectrum of activity similar to *T. harzianum*, but a slightly higher parasitism (*P* < 0.05) of *S. sclerotiorum* sclerotia (Fig. 2E).

### Taxonomy

**Fig. 3:**
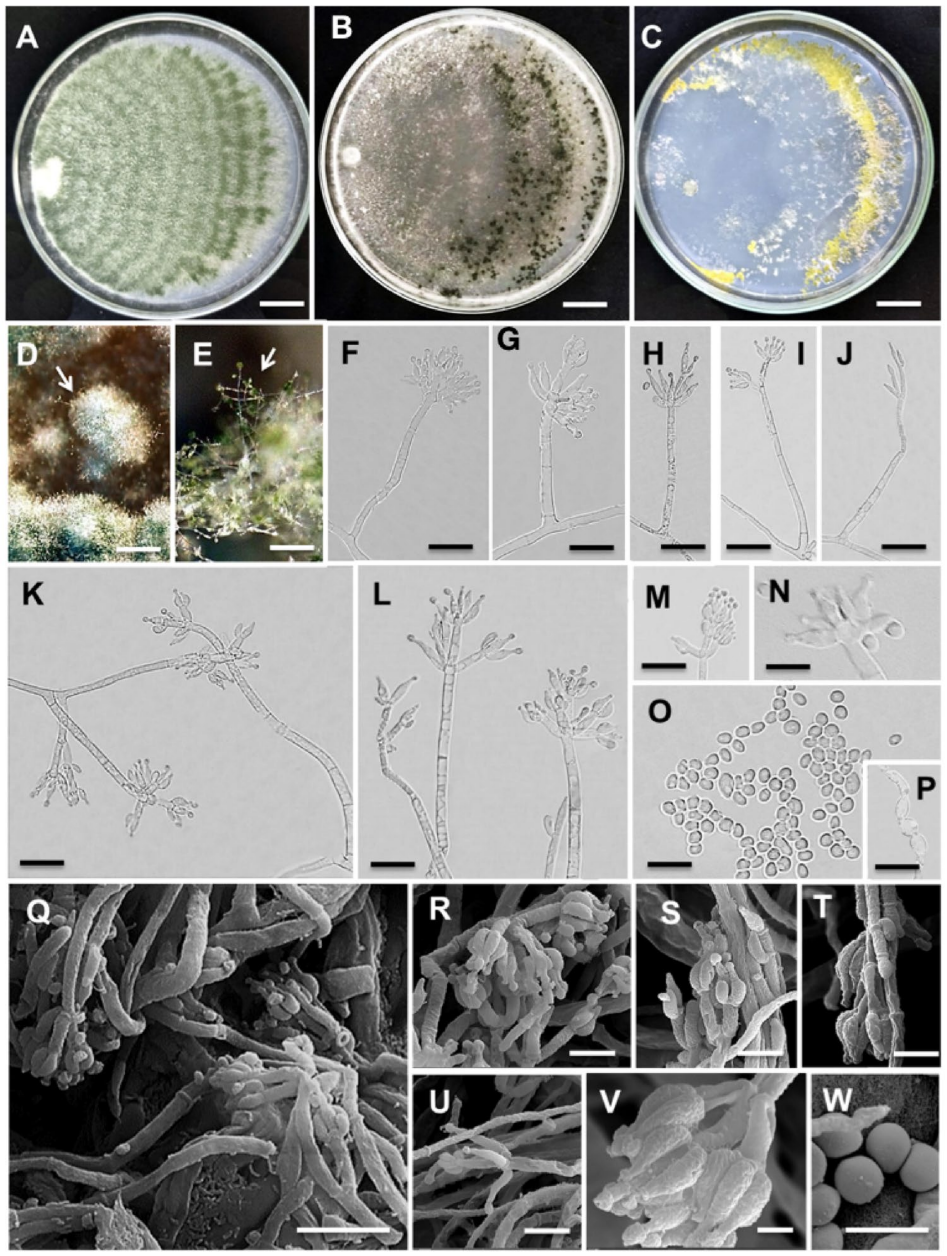
Morphological characteristics of *Trichoderma variabile* sp. nov. strain URMicro 11721. **A** Growth on PDA, **B** on SNA and **C** on CMD at 25 °C after 7 days. **D**,** E** Pustules and conidiophores in a stereomicroscope. **F-P** Conidiophores (F-L), phialides (K-N), conidia (N, O) and chlamydospores (P) in optical microscopy. **Q-W** Conidiophores (Q-U), phialides (R-V) and conidia (W) in scanning electron microscopy (SEM) micrographs. Scale bars: (**A-C**) = 14 mm; (**D**,** E**) = 5 mm; (**F-P**) = 5 μm; (**Q-W**) = 50 μm

*Trichoderma variabile* J. Rembinski, P.A.S. Marbach, J.T. De Souza, sp. nov. Figure 3.

MycoBank: MB 854568.

Type: Brazil, Bahia state, Valença municipality, Guaibim Restinga (13° 18’ 03” S/38° 57’ 57” W) from leaf litter samples, 10/11/2012, collected by J.P. Andrade. Holotype HURB 38020, metabolically inactive, dried culture on PDA deposited in the Herbarium of Recôncavo da Bahia Federal University (UFRB). Ex-type culture deposited at the culture collection of UFRB under number MON21 and at Federal University of Lavras under number URMicro 11721 at −80 °C. Accession numbers: *tef1* = OR762226; *rpb2* = OR762230; *cal1* = OR762235.

Etymology: variabile refers to the variable morpho-physiological characteristics of different strains of this species.

Additional strains examined: Brazil, Bahia, Valença, Guaibim Restinga (13° 18’ 03” S/38° 57’ 57” W), from leaf litter, 10/11/2012, J.P. Andrade. Strains: MON10A = URMicro 11718; MON10B = URMicro 11719 and MON19 = URMicro 11720 preserved in glycerol at −80 °C in the collections of Federal University of Recôncavo da Bahia (Bahia state) and Federal University of Lavras (Minas Gerais state).

Description: Conidiophores verticillium- and pachybasium-like, solitary, sometimes ramified and formed on sparse mycelia. Phialides in asymmetrical arrangement, varying from 1 to 3, lageniform, ampulliform and subulate, measuring (7.5–)8–16(–20) µm long, 2–3.5(–4) µm at the widest point, L/W ratio 3.5–8(–10), 1.5–2 μm (*n* = 50) at the base, surging from a cell 2–3 μm (*n* = 50). Conidia ellipsoidal and globose, smooth, varying from pale to dark green, measuring (2–) 3.5–7 × 2–4.5 μm (*n* = 50), L/W ratio (0.77)1–3.5. Chlamydospores 3–7 × 4–7 μm (*n* = 50), abundant, globose and suboval. Teleomorph not observed.

Culture characteristics: colony growth on SNA at 25 °C after 72 h, radius 35 mm, at 35 °C, 30 mm and at 15 °C, 25 mm. Mycelium covers the plate in 7 d at 25 °C. Colony initially hyaline turning dark green with conidiation, which starts at 96 h. Pustules distant from the inoculation point, mostly at the periphery of the plate (Fig. 3B), solitary to aggregated, sparse and circular, initially white, turning light green and later dark green, 5–10 mm. No distinct odor noticed. No pigment observed. On PDA at 25 °C after 72 h, colony radius 43 mm, at 35 °C, 38 mm, at 15 °C, 29 mm. Mycelium covers the plate in 7 d at 25 °C. Colonies are smooth, dense and white, turning green at conidiation, color is lighter when compared to SNA. Conidiation begins 96 h after inoculation, in concentric rings without forming pustules, directly on aerial mycelia (Fig. 3A). Light coconut odor noticed. No pigment observed. On CMD at 25 °C after 72 h, colony radius 32 mm; at 35 °C, 28 mm, at 15 °C, 26 mm. Mycelium covering the plate in 7 d at 25 °C. Colonies initially white, turning yellow and later on light green. Conidiation begins 96 h after the inoculation, on pustules distant from the inoculation point, produced in concentric rings at the plate periphery (Fig. 3C). No distinct odor noticed. No pigment observed.

Distribution: Only known from Brazil, Valença, Guaibim Restinga.

Notes: The species *T. variabile* fell in a well-supported cluster, separated from its closest relatives (Fig. 1). *Trichoderma variabile* could not be distinguished from its closest relatives, *T. confluens*, *T. decipiens* and *T. applanatum* on the basis of conidia and phialide morphology as well as in optimal growth temperatures [31–35; Table S2]. However, the growth rate on PDA at 25 °C differed among these species. *Trichoderma variabile* filled the plate in 7 days, whereas *T. confluens* did it in 3–4 days and the other two species, *T. applanatum* and *T. decipiens* were similar, taking 10–12 days (Table S2). *Trichoderma applanatum* was the only one with hyaline conidia and *T. decipiens* the only one producing a pinkish white pigment in the medium, whereas the other species, including *T. variabile*, did not release any pigment in the culture medium (Table S2).

**Fig. 4:**
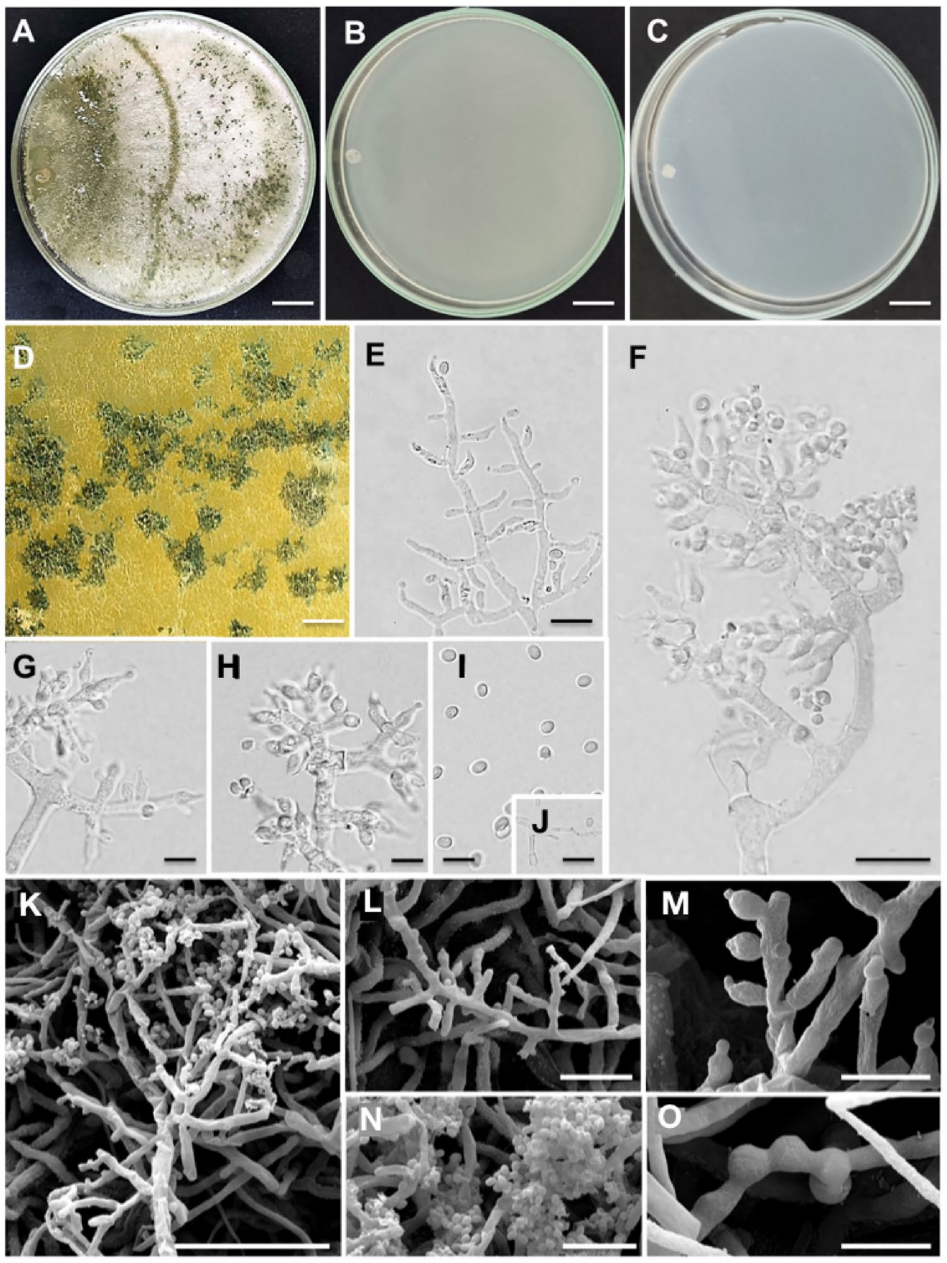
Morphological characteristics of *Trichoderma littericola* sp. nov. strain URMicro 11723. **A** Growth on PDA, **B** on SNA and **C** on CMD at 25 °C after 7 days. **D** Pustules on PDA under a stereomicroscope. **E-J** Conidiophores (E-H), phialides (E-H), conidia (I) and chlamydospores (J) in optical microscopy. **K-O** Conidiophores (K, L), phialides (L, M), conidia (K, M, N) and chlamydospores (O) in scanning electron microscopy (SEM) micrographs. Bars: **(A-C)** = 14 mm; **(D)** = 5 mm; **(E-J)** = 5 μm; **(K-O)** = 50 μm

*Trichoderma littericola* J. Rembinski, P.A.S. Marbach, J.T. De Souza, sp. nov. Figure 4.

MycoBank: MB 854567.

Type: Brazil, Bahia state, Guaibim Restinga (13°18’36” S/38° 58’ 13” W), from leaf litter, 02/10/2012, P.A.S. Marbach. Holotype HURB 38021, metabolically inactive, dried culture on PDA deposited in the UFRB herbarium. Ex-type culture URMicro 11723 deposited in the culture collection of Federal University of Lavras and at Federal University of Recôncavo da Bahia under number MTS13 at −80 °C. Accession numbers: *tef1* = OR762227; *rpb2* = OR762231; *cal1* = OR762236.

Etymology: littericola is a reference to leaf litter, the isolation source of this species. Description: Conidiophores trichoderma-like, formed on aerial mycelium and on small pustules (2–5 mm). Conidiophores typically with a central axis from which phialides are formed in vertexes and/or solitary. Phialides (3–)4.2–9(–10) µm long, (2–)2.2–3.8(–4) µm at the widest point (*n* = 50), L/W ratio (1.8)2–2.8(6), (1–)1.5–2.2(− 3) µm at the base, originating from a cell (1.4–)2–3(–3.5) µm of width, typically lageniform to ampulliform in the aerial mycelium. Conidia 2–3.5(–5) × 1.7–3 μm, L/W ratio (0.66)1.16–1.35(1.6) (*n* = 50), ellipsoidal and globose, smooth, light to dark green. Chlamydospores 7–10 × 3–5 μm (*n* = 50), globose to ovoid, abundant, intercalary and terminal. Teleomorph not observed.

Culture characteristics: optimum growth temperature on PDA, SNA and CMD, 25 °C. On PDA after 72 h, 90 mm at 15, 25 and 35 °C. Colonies were white transitioning to yellow and finally olive green and dark green. Pustules, small (3–7) mm, sparse, observed in the whole plate. On SNA after 72 h, 90 mm at 15, 25 and 35 °C. Colonies hyaline, difficult to observe (Fig. 4B). No pustules formed. On CMD after 72 h, 90 mm at 15, 25 and 35 °C. Colonies hyaline, sparse, without the formation of pustules, culture medium with a pallid yellow olivaceous pigment. Indistinct odor detected.

Distribution: Only known from Brazil, Valença, Guaibim Restinga.

Notes: *Trichoderma littericola* has shown fast growth at 35 °C and yellow pigment in the culture medium (PDA), and these are common characteristics in representatives of the *Longibrachiatum* clade [31]. The morphological characteristics, including the dimensions of the phialides, conidia and chlamydospores did not differentiate *T. littericola* from its closely-related species, *T. pinnatum* and *T. orientale* [31, 32]. However, chlamidospores were not observed in cultures of *T. pinnatum*, separating this species from *T. littericola* and *T. orientale*. Pustules were small (0.25–1 mm) in cultures of *T. pinnatum*, but these structures were not produced by *T. littericola* on SNA, differentiating these two species. The growth rate of *T. littericola* was the fastest, followed by *T. pinnatum* and *T. orientale*, although the temperature ranges were slightly different. *Trichoderma littericola* filled PDA and SNA plates in 3 days, whereas *T. pinnatum* took 3 days to fill PDA plates and 4 days to fill SNA plates and *T. orientale* filled both PDA and SNA plates in 4 days (Table S2).

*Trichoderma nordicum* G.Z. Zhang [15], MycoBank: MB 8212301. Accession numbers: *tef1* = MH287501; *rpb2* = MH287502.

Synonym: *Trichoderma nigricans* C.L. Zhang [33], MycoBank: MB 845506. Accession numbers: *tef1* = OP357974; *rpb2* = OP357959.

**Fig. 5:**
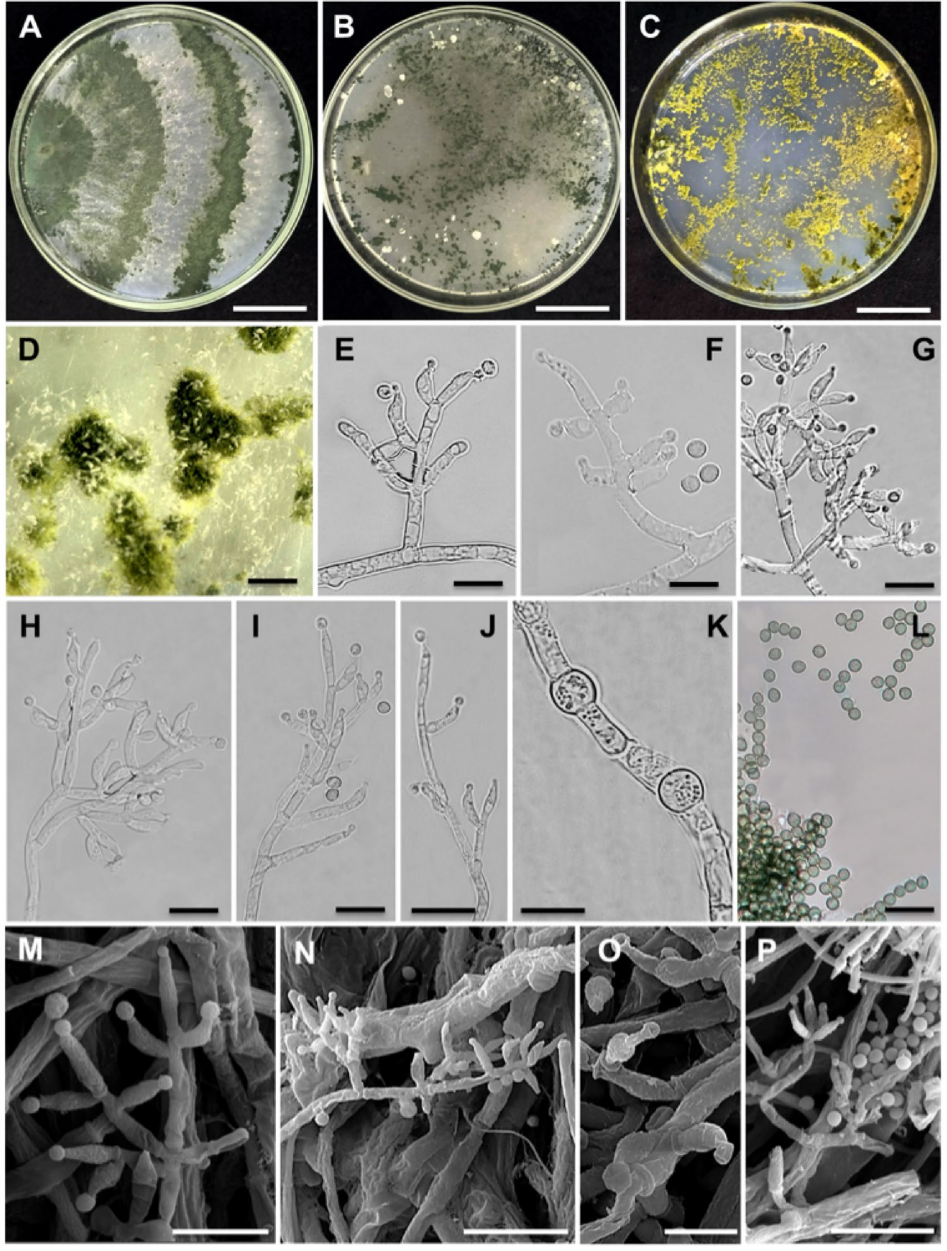
Morphological characteristics of *Trichoderma nordicum* strain URMicro 11722. **A** Growth on PDA, **B** on SNA and **C** on CMD at 25 °C after 7 days. **D** Pustules and conidiophores under a stereomicroscope. **E-L** Conidiophores (E-J), phialides (G-I), conidia (F, I, L) and chlamydospores (K) in optical microscopy. **M-P** Conidiophores (M, N, P), phialides (M-P) and conidia (M, N, P) in scanning electron microscopy (SEM) micrographs. Bars: (**A-C**) = 23 mm; (**D**) = 5 mm; (**E-L**) = 5 μm; (**M-P**) = 50 μm

Illustrations and descriptions: [15, 33]; Fig. 5; Tables S3 and S4.

Strain examined: Brazil, Amazonas, Manaus (3° 05’ 28” S/59° 59’ 36” W), soil, 12/10/2012. J.T. De Souza. Culture URMicro 11722. Accession numbers: *tef1* = HG325833; *rpb2* = OR762232.

Description: Conidiophores in a main axis, with lateral branches frequently at right angles. Phialides solitary or 2–3 in a spiral or cruciform pattern. Micromorphology of phialides, conidia, chlamydospores and culture characteristics in comparison with the Chinese strains are shown in Table S4. Cultures without any noticeable pigment or distinct odor.

Distribution: Asia (China, India, Sri Lanka, Taiwan), Americas (Brazil, Mexico, USA) (Table S3).

Notes: *T. nordicum* [15] and *T. nigricans* [33] are genetically and morphologically indistinguishable (Fig. S4; Table S4) and therefore should be synonymized under *T. nordicum*, according to the priority of publication. Strain URMicro 11722 of *T. nordicum* differs from the described Chinese strains by growing faster at 35 °C, but is similar in the other micromorphological characteristics (Table S4). Other sequences deposited in the database and misidentified, mainly as *T. atroviride* and as *Trichoderma* sp. were analyzed and correctly identified as *T. nordicum* (Table S3; Fig. S4).

## Discussion

In this study, two novel species of *Trichoderma* were described, *T. variabile* and *T. littericola*, and a third species, *T. nordicum*, was illustrated and *T. nigricans* was synonymized with it on the basis of taxonomic priority. The antagonistic potential of these *Trichoderma* species was tested in vitro, considering three mechanisms of activity, secretion of diffusible and volatile compounds, and parasitism of sclerotia. The results of this study extended our knowledge on the diversity of *Trichoderma* in the Restinga environment and point out for the potential application of the studied species in the biocontrol of sclerotia-producing plant pathogens.

*Trichoderma* is a hyperdiverse genus, with approximately 400 species described worldwide in 2021 [13, 34]. In this study we used sequences of *tef1*, *rpb2* and *cal* genes to infer phylogenies and to describe two novel *Trichoderma* species. The best marker to identify *Trichoderma* at the species level in terms of discriminatory power and representativeness in public databases is *tef1* [13, 14]. This marker is followed by *cal* in discriminatory power, but it unfortunately has a poor representativeness in public databases. On the other hand, *rpb2* has a good representativeness and ranks third in terms of discriminatory power [14]. Fragments of the ITS region of the rDNA have been recommended in *Trichoderma* phylogenies [13]. However, this region has no discriminatory power to distinguish *Trichoderma* species and is commonly not included in the description of novel species [14, 15, 17, 22, 31, 33, 35–37]. Due to the reasons outlined above, we did not determine the ITS sequences for the novel species that are being described in this study.

Studies showing the diversity of *Trichoderma* in natural and agricultural areas [37, 38], certain environments, such as the Restinga, are not well represented. The Restinga is an endangered ecosystem due to its exposure to disorganized economical exploitation and human occupation, and rising sea levels attributed to global warming [39]. The Restinga ecosystem harbors a diverse fungal community that may be exploited in biotechnological applications, such as the biocontrol of fungal pathogens. Our research group is systematically sampling the Restinga ecosystem in Brazil to study the fungal communities occurring in this environment, with emphasis on the genus *Trichoderma* with the objective of ex-situ preservation and application in the control of plant diseases, especially the ones caused by sclerotia-producing soilborne pathogens.

The phylogenetic analysis performed in this study lead to the description of two novel species and also to the invalidation of the species *T. nigricans* and its synomyzation with *T. nordicum*. One of the problems frequently encountered in fungal taxonomy is the incompleteness of sequence databases and the lack of standardization of the sequenced regions. In this case study, *T. nordicum* was described by [15], but the sequences of the type strain were not annotated as such in the database, leading Zhao et al. [33] to describe *T. nigricans*. To avoid this type of confusion, it is advisable that all authors make sure the species published by them are fully annotated in sequence databases.

*Trichoderma nordicum* was previously known only from China [15], but our phylogeographic analysis of deposited sequences revealed that this species is distributed in Asia and the Americas (Table S3). Besides that, *T. nordicum* was found in very diverse environments and substrates such as soil, dead wood, algae, leaves cut by ants, from healthy plant tissues, manatee skin and turtle shell (Table S3), pointing out to the high degree of opportunism of this species.

The newly described species were isolated from soil collected at the Guaibim Restinga ecosystem. This ecosystem is subjected to extreme conditions, such as high temperature variation, UV radiation and strong winds. The strain of *T. nordicum* we examined was obtained from the Amazoniam region, which is also exposed to high temperatures. The adaptation to high temperatures of all the studied strains might, at least in part, explain the fast growth rate they had on culture media, with exception of *T. variabile*, which had a slower growth rate. It appears that, at least in some cases, there is an association between antagonistic activity and growth rate. There is evidence that temperature and the previous adaptation of the *Trichoderma* strains influence their activity in mycoparasitism and induction of resistance [40–42]. In fact, the strains of *T. variabile* examined in this study showed a slower growth rate at high temperatures and no inhibition of *S. sclerotiorum* with diffusible and volatile compounds (Fig. 2). It would be interesting to study the correlation between adaptation to high or low temperatures, for example, and antagonistic activity in the same background species. In this regard, testing Brazilian and Chinese strains of *T. nordicum*, adapted to annual average temperatures of 25–27 °C in Bahia and Amazonas states (Table S3), Brazil and 12–13 °C in Beijing and Shandong province, China (https://en.climate-data.org/) in sclerotia parasitism in vitro and in field trials would bring important knowledge. Most *Trichoderma* species are well adapted to grow at temperatures around 25 °C [43, 44]. There are, however, plant pathogens, such as *S. sclerotiorum* and *S. cepivora* that cause damage in the field at temperatures lower than 18 °C [45, 46]. In these cases, it is important to select strains adapted to grow, sporulate and survive at these temperatures.

*Sclerotinia sclerotiorum* produced mycelia and sclerotia that were more resistant to the *Trichoderma* strains than *Stromatinia cepivora*, although both pathogens are in the same family, *Sclerotiniaceae*. The size, structure and resistance of the sclerotia produced by these fungi differ considerably. While sclerotia produced by *S. cepivora* are 0.2–0.5 mm in diameter, have a thin rind of 1–2 cell layers and can stay viable for up to 20 years in soil, the ones produced by *S. sclerotiorum* are 2–10 mm in diameter, 2–3 cell layers thick and survive in soil on average for 3 years [47]. The reasons for this differential susceptibility shown by the two pathogens to *Trichoderma* are still unknown, although there are some indications on the sclerotial phase of these fungi. Sclerotia of *Sclerotinia* spp. were shown to secrete metabolites [48], and some metabolites have antimicrobial activity, such as phenolic and ferulic acids [49]. However, further studies are necessary to corroborate this hypothesis.

The species *T. littericola* and *T. nordicum* showed the highest potential to inhibit and parasitize sclerotia of *S. sclerotiorum* and *S. cepivora*, surpassing in most cases the *T. harzianum* strain used in our comparisons, which is the active ingredient of a commercial product. It is known that *Trichoderma* species employ several mechanisms of activity, frequently synergistically, depending on the system [50]. In our study, although we did initial tests on three mechanisms of activity commonly employed by *Trichoderma*, our main targets are the sclerotia and therefore, we consider mycoparasitism as the most important mechanism to decrease the concentration of these structures in soil. We are aware that the relationship between in vitro and in vivo experiments is poor [51], and therefore it is imperative to perform experiments *in planta*, preferentially in field trials.

All the *Trichoderma* strains studied were able to produce chlamydospores abundantly, especially on SNA at 25 °C. This opens up the possibility of producing inocula of these strains to apply as biocontrol agents based on these propagules as they are more resistant to adverse conditions than conidia [52]. Chlamydospores are hard to obtain in high concentrations and currently there is a lack of ideal fermentation technologies to produce them efficiently [52]. Understanding the genetic mechanisms that lead to their formation will help improve mass production of these resistance structures and optimize biocontrol formulations.

## Conclusions

Two new species of *Trichoderma*, *T. variabile* and *T. littericola*, were described in this study, contributing to increase our knowledge of the fungal diversity in the Restinga ecosystem. Additionally, the potential antagonism in vitro of these newly described species was demonstrated against two sclerotia-producing pathogens of worldwide importance. The species *T. nordicum* was reported from Brazil and *T. nigricans* was shown to be an invalid species name. The taxonomical novelties reported herein advance our knowledge on the diversity of the genus *Trichoderma*.

## Supplementary Information

Below is the link to the electronic supplementary material.

Supplementary Material 1

## Data Availability

The alignments used to infer the phylogenetic trees described in this study and the raw data on antagonism were deposited with the Zenodo repository and are available at 10.5281/zenodo.18215935.
