# Supplementary material for: Two Novel Trichoderma Species and their Antagonistic Activity against Sclerotia-producing Plant Pathogens
Source: Curr Microbiol. 2026 Mar 3;83(4):217. doi: 10.1007/s00284-026-04769-6 (PMC12957131; doi:10.1007/s00284-026-04769-6)
Supplement: Supplementary file 1 — Supplementary Material 1 [file 284_2026_4769_MOESM1_ESM.docx]

# Supplementary Material

# Two novel *Trichoderma* species and their antagonistic activity against sclerotia-producing plant pathogens

Jessica Rembinski, Lucas S. Sales, Phellippe A. S. Marbach, Jorge T. De Souza

**
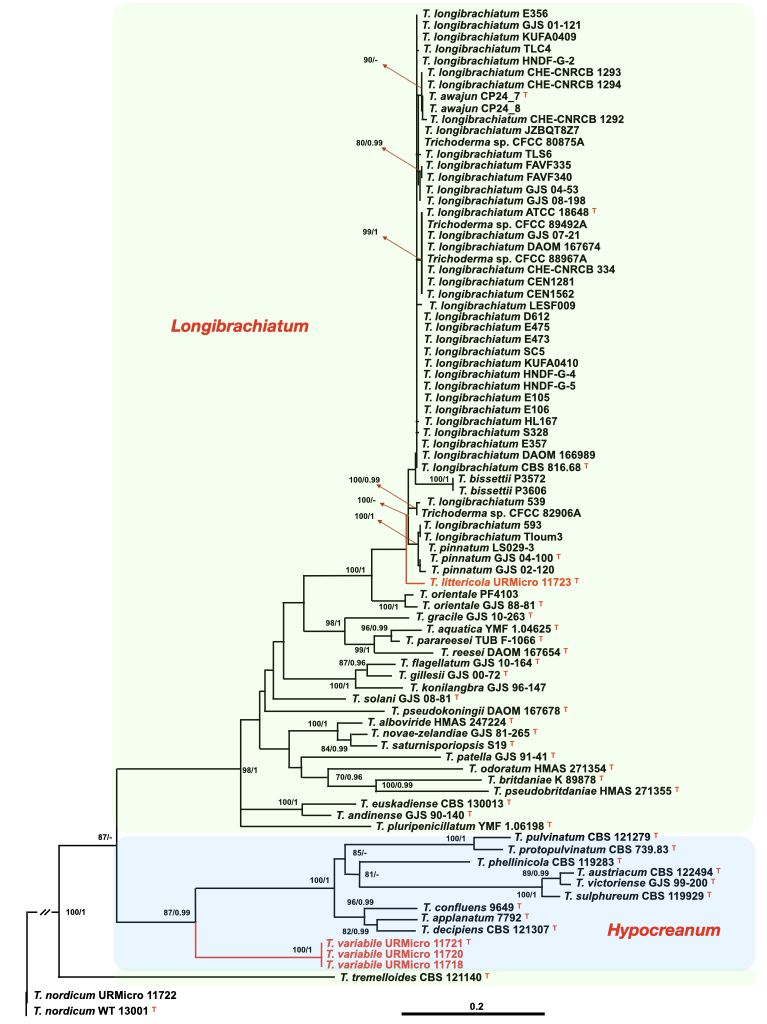
**

**Fig. S1.** Phylogenetic tree inferred with the maximum likelihood (ML) and Bayesian methods with 617 bp of aligned sequences of the *tef1* gene and the evolutionary model TIM2+I+G. The species *Trichoderma nordicum* strains URMicro 1172 and WT 13001 were used as outgroups. The numbers on the nodes represent the bootstraps (1000 resamplings) in ML analysis followed by the posterior probabilities (PP) in the Bayesian analysis. A dash means that the bootstrap or PP values are not significant according to the following reference values for significance: >70% for bootstrap and >0.95 for PP. The species described in this study are shown in red. The clades of *Trichoderma* are shown in different background colors. The type strains of each species are indicated with a superscript T. The scale indicates the number of substitutions per site.

**
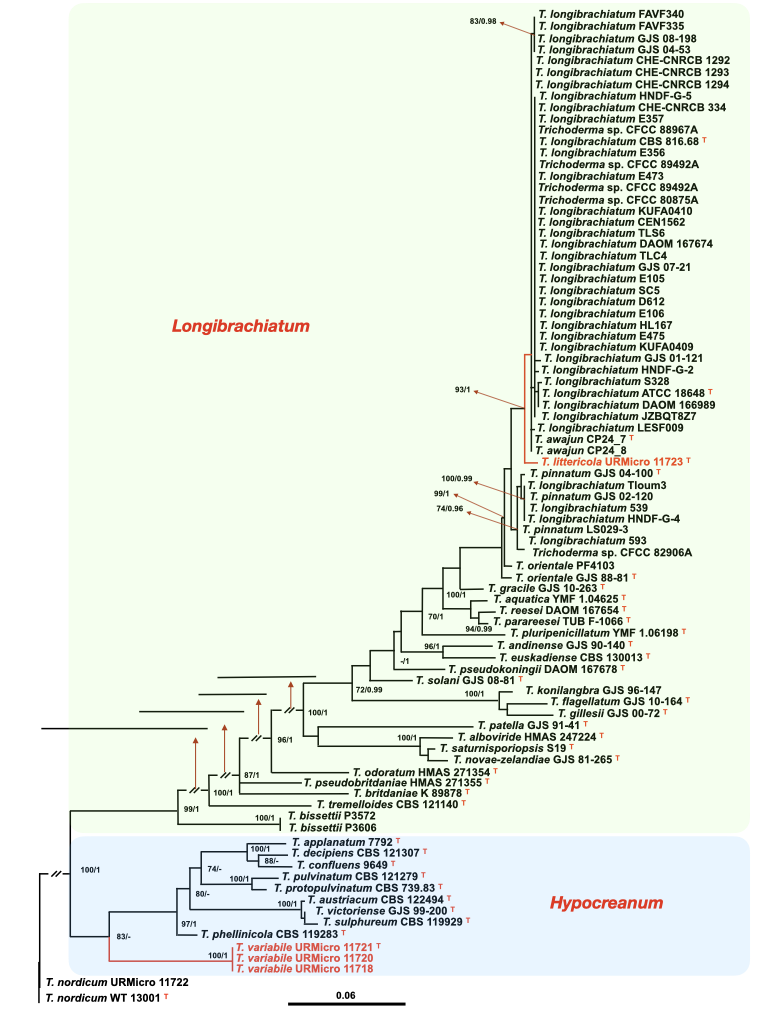
**

**Fig. S2.** Phylogenetic tree inferred with the maximum likelihood (ML) and Bayesian methods using 544 bp of aligned sequences of the *rpb2* gene and the evolutionary model TrN+I+G. The species *Trichoderma nordicum* strains URMicro 1172 and WT 13001 were used as outgroups. The numbers on the nodes represent the bootstraps (1000 resamplings) in ML analysis followed by the posterior probabilities (PP) in the Bayesian analysis. A dash means that the bootstrap or PP values are not significant according to the following reference values for significance: >70% for bootstrap and >0.95 for PP. The species described in this study are shown in red. The length of shortened branches is indicated with red arrows. The clades of *Trichoderma* are shown in different background colors. The type strains of each species are indicated with a superscript T. The scale indicates the number of substitutions per site.

**
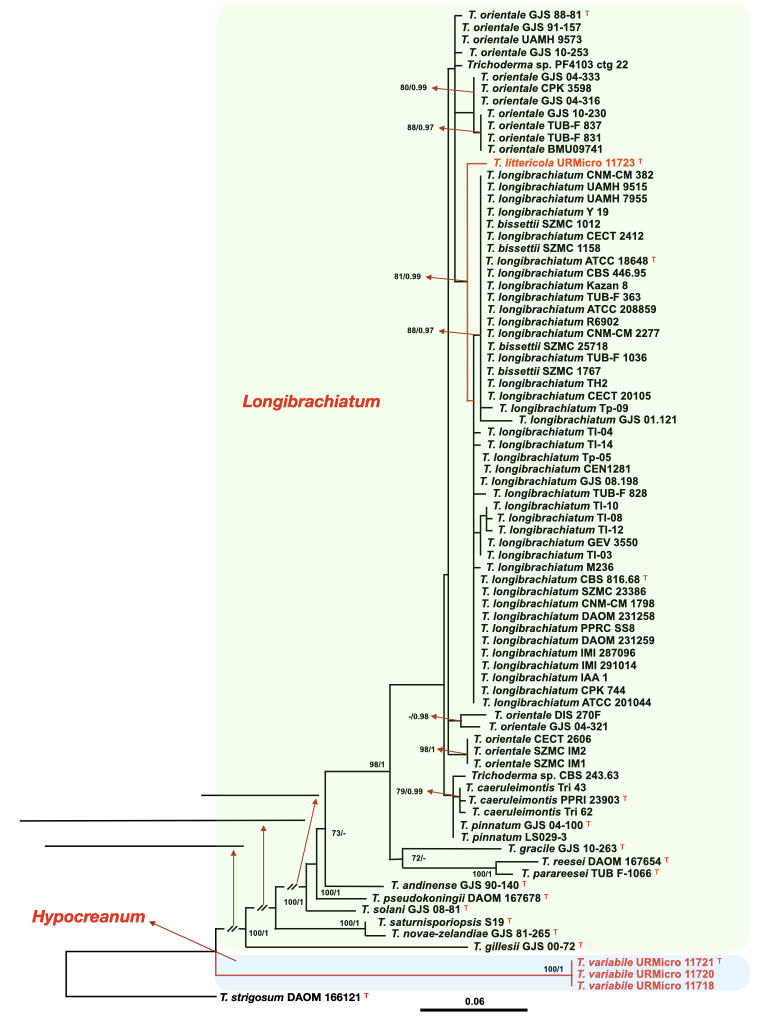
**

**Fig. S3.** Phylogenetic tree inferred with the maximum likelihood (ML) and Bayesian methods using 369 bp of aligned sequences of the *cal1* gene and the evolutionary model TIM1ef+I. The species *Trichoderma nordicum* strains URMicro 1172 and WT 13001 were used as outgroups. The numbers on the nodes represent the bootstraps (1000 resamplings) in ML analysis followed by the posterior probabilities (PP) in the Bayesian analysis. A dash means that the bootstrap or PP values are not significant according to the following reference values for significance: >70% for bootstrap and >0.95 for PP. The species described in this study are shown in red. The length of shortened branches is indicated with red arrows. The clades of *Trichoderma* are shown in different background colors. The type strains of each species are indicated with a superscript T. The scale indicates the number of substitutions per site.

**
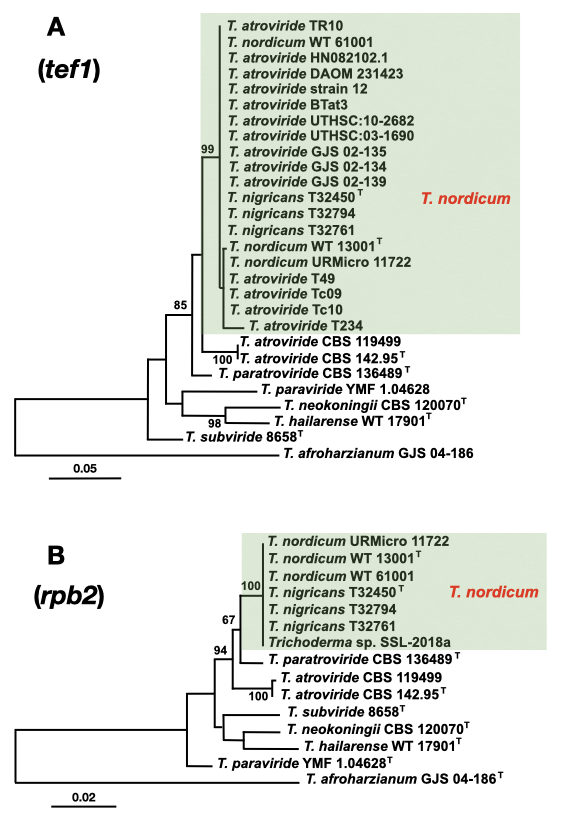
**

**Fig. S4.** Phylogenetic trees inferred with the maximum likelihood (ML) method with sequences of *tef1* **(A)** and *rpb2* **(B)** genes. The ML trees were inferred with 444 bp of *tef1* and 813 bp of the *rpb2* gene and the evolutionary model GTR + G. The species *T. afroharzianum* was used as an outgroup. The bootstrap numbers (1000 resamplings) higher than 70% are shown on the appropriate nodes. The type strains of each species are indicated with a superscript T. The scale indicates the number of substitutions per site.

**Table S1.** Primers used for the amplification and sequencing of the marker genes employed in our phylogenetic analyses.

| **Locus** | **Definition** | **Primer name** | **Sequence (5’ – 3’)** | **Amplified**  **fragment** | **Annealing**  **temperature** | **Reference^a^** |
| --- | --- | --- | --- | --- | --- | --- |
| *tef1* | Translation elongation factor 1 alpha | TEF1-728 F | CAT CGA GAA GTT CGA GAA GG | ~1200 bp | 58 °C | 20 |
|  |  | TEF1-LLErev | AAC TTG CAG GCA ATG TGG |  |  | 22 |
| *rpb2* | RNA polymerase subunit 2 | fRPB2-5F | GAY GAY MGW GAT CAY TTY GG | ~1000 bp | 55 °C | 21 |
|  |  | fRPB2-7CR | CCC ATR GCT TGY TTR CCC AT |  |  | 21 |
| *cal1* | Calmodulin | CAL-228F | GAG TTC AAG GAG GCC TTC TCC C | ~500 bp | 55 °C | 20 |
|  |  | CAL-737R | CAT CTT TCT GGC CAT CAT GG |  |  | 20 |

^a^ See the reference list of this publication.

**Table S2.** Morphological and physiological characteristics of the novel *Trichoderma* species being described in this study and their closest relatives. Microscopic measurements of phialides, conidia and chlamydospores were done in SNA medium.

| **Species/**  **Type strains** | **Phialides** | **Conidia** | **Growth** | **Chlamydospores (Chl)/ pustules (Pst)/ pigments (Pgm)** | **Reference** |
| --- | --- | --- | --- | --- | --- |
| ***T. variabile*** sp. nov.  URMicro 11721 | (7.5–)8–16(–20) um long  2–3.5(–4) um at widest point  3.5–8(–10) L/W  1.5–2 um base  2–3 um cell | (2–)3.5–7 × 2–4.5 um  (0.77)1–3.5 L/W  Green/smooth | **PDA:** (72 h; 9 cm plates):  25 °C: 43 mm, fills plate in 7 d  15 °C: 38 mm  35 °C: 29 mm  **SNA:** (72 h; 9 cm plates):  25 °C: 35 mm  15 °C: 25 mm  35 °C: 30 mm | **Chl**: 3–7 × 4–7 um  **Pst**: 5–10 mm  **Pgm**: no pigments | This study |
| *T. confluens*  HMAS 244995 | 10.5–19(–26) um  2–3(–4) um widest point  (3–)3.5–8(–9) L/W | (3.5–)4–7.5(–8) × (2-) 2.5–3.5(–4.5) um  (3–)3.5-8(–9) L/W | **PDA:** (72 h; 9 cm plates):  25 °C: fills plate in 3–4 d  **SNA:** (72 h; 9 cm plates):  25 °C: 45–51 mm | **Chl**: (5.5–)6–16(–20) × (5–)5.5–14.5(–15) um  **Pst**: no information  **Pgm**: no pigments | [33] |
| *T. applanatum*  HMAS 245081 | (10–)18–42(–60) um  (3–)4–6(–7) um widest  (3–)5–11(–15) L/W  (1.5–)3.5–5(–6.5) um base | 4–9.5 × 2.5–3.5 um  1.7–3 L/W  Hyaline/smooth | **PDA:** (72 h; 9 cm plates):  25 °C: 21–31 mm, fills plate in 10–12 d  **SNA:** (72 h; 9 cm plates):  25 °C: 8–17 mm, fills plate in 12–14 d | **Chl**: no information  **Pst**: no information  **Pgm**: no pigments | [34] |
| *T. decipiens*  CBS 121307 | (9.5–)13–25(–36) um long  (1.5–)2.4–3(–3.4) um widest | (3–)4.4–6.7(–11.6) × (1.9–)2.5–3.5(–5) um | **PDA:** (72 h; 9 cm plates):  25 °C: fills plate in 10 d  **SNA:** (72 h; 9 cm plates):  25 °C: fills plate in 19 d | **Chl**: no information  **Pst**: no information  **Pgm**: pinkish white | [35] |
| ***T. littericola*** sp. nov.  URMicro 11723 | (3–)4.2–9(–10) um long  (2–)2.2–3.8(–4) um widest  (1.8)2–2.8(6) L/W  (1–)1.5–2.2(–3) um base  (1.4–)2–3(–3.5) um cell | 2–3.5(–5) × 1.7–3 um  (0.7)1.2–1.4(1.6) L/W  Green/smooth | **PDA:** (72 h; 9 cm plates):  15, 25, 35 °C: 90 mm  **SNA:** (72 h)  15, 25, 35 °C: 90 mm | **Chl**: 7–10 × 3–5 um  **Pst**: not formed on SNA  **Pgm**: yellow | This study |
| *T. pinnatum*  CBS 131292 | (4.2–)5.5–9(–12) um long  (2–)2.5–3.5(–4.2) um widest  (1.3–)1.5–3.5(–5) L/W  (1.2–)1.5–2.2(–2.7) base  (1.7–)2–3(–4) um cell | (2.2–)2.5–3.5(–5) × (1.7–)2.5–3(–3.5) um  (1.2–)1.3–1.7(–1) L/W  Green/smooth | **PDA:** (72 h; 9 cm plates):  30–35 °C: 90 mm  **SNA:** (96 h; 9 cm plates):  25–30 °C: 90 mm | **Chl**: not observed  **Pst**: 0.25–1 mm (SNA)  **Pgm**: pale yellow | [36] |
| *T. orientale*  CBS 130428 | (3.5–)6.2–10.5(–15.7) um long  (2–)2.5–3.7(–4.5) um widest  (1.3–)1.6–3.8(–7.7) L/W  (1–)1.7–2.7(–3.5) base  (1.5–)2.5–4(–5.5) um cell | (3.2–)3.7–6.2(10.5) × (2–)2.5–3.5(–5.2) um  (1.1–)1.3–2.5(4.9) L/W  Green/smooth | **PDA:** (96 h; 9 cm plates):  25–35 °C: 90 mm  **SNA:** (96 h; 9 cm plates):  30–35 °C: 90 mm | **Chl**: (4.5–)6.2–9(–14) um  **Pst**: not measured  **Pgm**: yellow; not at 35 °C | [36] |

**Table S3.** Accession numbers of strains used in this study to identify the species *T. nordicum* in deposited sequences. The current names of sequences deposited in public databases were defined with phylogenetic analyses.

| **Species name in GenBank** | **Strain** | **Origin** | **Isolation** | **Accession number** | | | **Current name** |
| --- | --- | --- | --- | --- | --- | --- | --- |
|  |  |  |  | ***tef1*** | ***rpb2*** | |  |
| *T. afroharzianum* | CBS 124620 ^T^ | Peru | Mycoparasite *M. roreri* | FJ463301 | | FJ442691 | *T. afroharzianum* |
| *T. hailarense* | WT 17901 ^T^ | China | Soil | MH287505 | | MH287506 | *T. hailarense* |
| *T. neokoningii* | CBS 120070 ^T^ | Peru | Mycoparasite *M. roreri* | KJ665620 | | KJ665318 | *T. neokoningii* |
| *T. atroviride* | CBS 142.95 ^T^ | Slovenia | Beetle gallery | AY376051 | | EU341801 | *T. atroviride* |
| *T. atroviride* | CBS 119499 | Austria | Bark and wood | FJ860611 | | FJ860518 | *T. atroviride* |
| *T. paraviride* | YMF 1.04628 ^T^ | China | Root endophyte | MK775508 | | MK775513 | *T. paraviride* |
| *T. subviride* | HMAS 273761 ^T^ | China | Twigs | KU529131 | | KU529142 | *T. subviride* |
| *T. paratroviride* | CBS 136489^T^ | Spain | Tree branch | KJ665627 | | KJ665321 | *T. paratroviride* |
| *T. nordicum* | URMicro 11722 | Brazil, Amazonas | Dead wood | HG325833 | | OR762232 | *T. nordicum* |
| *T. nordicum* | WT 13001 ^T^ | China | Soil | MH287501 | | MH287502 | *T. nordicum* |
| *T. nordicum* | WT 61001 | China | Soil | MH287503 | | MH287504 | *T. nordicum* |
| *T. nigricans* | T32450 | China | Maize soil | OP357973 | | OP357958 | *T. nordicum* |
| *T. nigricans* | T32794 | China | Maize soil | OP357975 | | OP357960 | *T. nordicum* |
| *T. nigricans* | T32781 = CGMCC40314 ^T^ | China | Peach soil | OP357974 | | OP357959 | *T. nordicum* |
| *T. atroviride* | DAOM 231423 | Mexico | Soil | EU280002 | | - | *T. nordicum* |
| *T. atroviride* | T234 | Brazil | Unknown | MT681978 | | - | *T. nordicum* |
| *T. atroviride* | HN082102.1 | China | Algae | MW133238 | | - | *T. nordicum* |
| *T. atroviride* | T49 | Brazil | Unknown | MT681973 | | - | *T. nordicum* |
| *T. atroviride* | TR10 | India | Piper nigrum soil | LC800025 | | - | *T. nordicum* |
| *T. atroviride* | GJS 02-139 | Sri Lanka | Herbaceous stem | DQ307546 | | - | *T. nordicum* |
| *T. atroviride* | GJS 02-134 | Sri Lanka | Dead wood | DQ307547 | | - | *T. nordicum* |
| *T. atroviride* | GJS 02-135 | Sri Lanka | Dead tree | DQ307548 | | - | *T. nordicum* |
| *T. atroviride* | UTHSC:03-1690 | USA | Manatee skin | HG931221 | | - | *T. nordicum* |
| *T. atroviride* | UTHSC:10-2682 | USA | Turtle shell | HG931223 | | - | *T. nordicum* |
| *T. atroviride* | BTat3 | India | Clusterbean | OQ362336 | | - | *T. nordicum* |
| *T. atroviride* | 12 | Brazil | Leaf cut by ant | KT619055 | | - | *T. nordicum* |
| *T. atroviride* | Tc09 | Brazil, Bahia | Endophyte | KP890328 | | - | *T. nordicum* |
| *T. atroviride* | Tc10 | Brazil, Bahia | Endophyte | KP890329 | | - | *T. nordicum* |
| *Trichoderma* sp. | SSL-2018a | Taiwan | Unknown | - | | MF043189 | *T. nordicum* |

**Table S4.**  Comparison of the morphophysiological characteristics of Brazilian and Chinese strains of *Trichoderma nordicum*.

| **Characteristic** | ***T. nordicum***  **URMicro 11722**  **(This study)** | **T. *nordicum* WT 13001^T^**  **(Zhang et al. 2022)** | **T *nigricans* T32781^T^**  **(Zhao et al. 2023)** |
| --- | --- | --- | --- |
| **Origin** | Brazil, Amazonas, Manaus, soil | China, Beijing, Yu-yuan-tan Park, soil | China, Shandong, Dezhou city, peach rhizosphere soil |
| **Growth on PDA after 72 h** | 15 °C – 56 mm  25 °C – 90 mm (3 days)  35 °C – 90 mm | 25 °C – 67–71 mm  35 °C – did not grow | 25 °C – 90 mm  30 °C – 55–61 mm  35 °C – 16 mm |
| **Growth on SNA after 72 h** | 15 °C – 65 mm  25 °C – 90 mm (3 days)  35 °C – 90 mm | 25 °C – 21–24 mm  35 °C – did not grow | 25 °C – 50–70 mm  30 °C – 50–60 mm  35 °C – 50–60 mm |
| **Growth on CMD after 72 h** | 15 °C – 62 mm  25 °C – 90 mm (3 days)  35 °C – 90 mm | 25 °C – 68–71 mm  35 °C – did not grow | 25 °C – 90 mm  30 °C – 90 mm  35 °C – 20–22 mm |
| **Phialides** | (5–)6.2–11(–14) × (2–)2.5 – 3.2(–3.5) µm | (6.2–)7.2–10.3(–12.9) × (2.6–) 2.9–3.2(–3.4) μm | (4.7–)6.0–8.9(–12.1) × (2.5–)2.9–3.4(–4.5) μm |
| **L/W ratio at the base** | (1)1.5–2.3(–3) | (2.1–)2.4– 3.4(–4.3) (mean = 2.9) | (1.2–)1.8–2.9(–3.6) |
| **Conidia** | (3–)3.3–3.7(– 4) × (3–)3.2–3.5 (–3.7) µm | (4.1–)4.4–4.8(–5.0) × (4.0–) 4.1–4.4(–4.6) | (3.0–)3.2–3.6(–3.9) × (2.8–) 3.1– 3.4(–3.8) μm |
| **L/W ratio of conidia** | (0.9–)1–1. (–1.2) µm | 1.0–1.2 (mean = 1.1) | 1.0–1.1 (mean = 1.1) |
| **Chlamydospores** | 4–6 × 8–10 μm  Abundant | (8.7–)9.8 × 10.4(–12.5) μm Sometimes present | (7.2–)7.8–9.2(–10.1) × (6.1–)7.1–9.0(–9.7) μm (mean = 8.6 x 8.1 μm), Abundant |
